# Correcting zinc deficiency in nursing home residents: a randomized trial comparing 30 and 60 mg/day elemental zinc supplementation

**DOI:** 10.3389/fnut.2026.1935770

**Published:** 2026-09-30

**Authors:** Edwin F. Ortega, Jonathan H. Yao, Weimin Guo, Sarah K. Boehm, Elizabeth Ergil, Dayong Wu, Simin N. Meydani, Alexander Panda

**Affiliations:** 1 Nutritional Immunology Laboratory, JM USDA Human Nutrition Research Center on Aging, Tufts University, Boston, MA, United States; 2 Department of Microbiology and Immunology, University of California, San Francisco, San Francisco, CA, United States; 3 Translational Immunogenomics Laboratory, Department of Medical Oncology, Dana-Farber Cancer Institute, Boston, MA, United States; 4 Department of Pathology and Laboratory Medicine, Boston University Chobanian and Avedisian School of Medicine, Boston, MA, United States; 5 Friedman School of Nutrition Science and Policy, Tufts University, Boston, MA, United States; 6 Department of Immunology, School of Medicine, Tufts University, Boston, MA, United States

**Keywords:** double-blind randomized controlled trial, nursing home residents, older adults, zinc deficiency, zinc supplementation

## Abstract

**Introduction:**

Low serum zinc is associated with higher pneumonia incidence and illness duration compared to individuals with adequate serum zinc levels. However, the optimal and safe zinc supplementation dose to correct deficiency has not been established. This study aimed to determine the optimal and safe dose of elemental zinc supplementation to correct deficiency in older adults residing in nursing homes.

**Methods:**

Methods: We conducted a 12-month randomized, placebo-controlled, double-blind clinical trial in zinc-deficient nursing home residents, comparing supplementation of 30 and 60 mg/day elemental zinc versus placebo on zinc status and potential adverse effects (n = 26-35/group).

**Results:**

Both the 30 and 60 mg/day groups significantly increased serum zinc levels compared to placebo (*p* < 0.001). All participants in the 60 mg/day group reached the target threshold (> 70 μg/dL), with 100% achieving zinc adequacy by month 8. In contrast, only 46.4% (13 of 28) of participants in the 30 mg/day group reached this threshold, with the proportion plateauing at month 8. No participant in the placebo group reached zinc adequacy. No impact on copper status was detected. Although not powered for clinical outcomes, exploratory analyses showed participants in the placebo group reported significantly more coughing episodes, fevers, and chills throughout the study compared to those in 30 and 60 mg/d supplemented groups.

**Discussion:**

In zinc deficient older adults, 60 mg/day of elemental zinc effectively corrected deficiency with no adverse effects. These findings inform the dosing protocol for a fully powered randomized trial testing the effect of zinc supplementation on pneumonia incidence and outcomes in this population.

**Clinical trial registration:**

https://clinicaltrials.gov/, identifier NCT05527899.

## 1 Introduction

Pneumonia (PNA) remains a significant public health concern, particularly amongst older adults (1) living in nursing homes (NH) (2). In these settings, factors like limited mobility, inadequate nutriture, (3) and higher prevalence of chronic conditions (4), compounded with age-associated declines in physiological and immune function (5), increase susceptibility to respiratory infections. The economic impact of PNA is substantial, with treatment costs of $458/case in NH in the United States (6). While proactive measures, such as vaccination, improved public health practices, and routine health monitoring, can reduce PNA cases and severity of disease (7), these strategies alone may be insufficient (8). Targeted micronutrient supplementation represents a complementary approach that could play a vital role in bolstering immune defenses against respiratory infections in older adults resulting in less pneumonia.

Zinc (Zn) is one such micronutrient, well-established for its critical role in immune cell function and host defense against infection (9), and thus a candidate for reducing infection rates and lowering PNA-related hospitalizations. The relationship between Zn status and age-related immune decline has been studied extensively, including by the ZINCAGE consortium, which investigated the role of Zn in immunosenescence and “inflammaging” in older adults using fixed, low-dose supplementation (10). The present study addresses a distinct question: identifying the optimal dose of elemental Zn needed to correct confirmed Zn deficiency in a NH population, as a prerequisite for evaluating downstream clinical outcomes such as PNA risk. Previously, we reported that 29% of NH residents in and around the Boston area had low serum Zn levels (< 70 μg/dL), which was associated with significantly higher incidence and duration of PNA, increased antibiotic use, and overall mortality—associations that remained independent of age, albumin levels, other micronutrient levels, C-reactive protein (CRP), and other predisposing conditions (11). These observational findings are further supported by a small study conducted by Prasad et al. (12), in which older adults supplemented with 45 mg/d of Zn for 12 months exhibited lower incidence of all infections including respiratory illness. However, causality cannot be established from observational data, and a prospective clinical trial of much larger size is needed to determine whether correcting Zn deficiency can reduce PNA incidence in this population.

As a necessary first step toward that goal, our group conducted a preliminary randomized, double-blind, placebo-controlled trial in which NH residents with low serum Zn were supplemented with 30 mg/d of Zn gluconate for 3 months (13). Consistent with our previous study, 31 of 53 screened participants (58%) had low serum Zn concentrations at baseline, further demonstrating the high prevalence of Zn deficiency in this population. Although serum Zn levels improved in most participants, 42% of those who completed the study in the Zn group did not reach adequate serum Zn concentrations (≥ 70 μg/dL) by month 3. These participants had very low baseline serum Zn levels (mean ± SD of 53 ± 6 μg/dL, range of 44–60 μg/dL), suggesting that a subset of Zn-deficient older adults may require higher supplementation doses or longer intervention periods to reach adequacy.

To address this, we conducted a 1-year, randomized, double-blind, placebo-controlled study comparing supplementation with 30 and 60 mg/day of elemental Zn in Zn-deficient NH residents (NCT05527899). The primary outcome of this pilot study was the proportion of participants achieving serum Zn adequacy (≥ 70 μg/dL) for each dose. Secondary outcomes included adverse events, self-reported respiratory symptoms, and changes in clinical chemistries, including copper status. Intestinal absorption of copper (Cu) is inhibited by Zn. Thus, chronic intake of high doses of Zn may be associated with Cu deficiency (14). This concern is the main basis for the upper tolerable limit (UL) for Zn (15). However, this UL applies to individuals with adequate Zn status and does not preclude therapeutic use in Zn-deficient populations. The findings from this dose-finding trial are intended to inform the design and dosing protocol for a fully powered randomized trial to examine the effect of Zn supplementation on PNA incidence and outcomes in older adults residing in NH.

## 2 Materials and methods

### 2.1 Study design

A one-year long randomized, double-blind, placebo-controlled trial (RCT) was conducted to determine an effective and safe Zn supplementation dosage for elderly NH residents with low serum Zn levels (< 70 μg/dL), this threshold is consistent with that recommended by Institute of Medicine (IOM), panel on micronutrients (15) and others (16). Two Zn dosages, 30 or 60 mg/d, were tested against a placebo control. The primary objectives were to establish the optimal dose of Zn supplementation required to achieve serum Zn adequacy. The secondary objectives were to assess adverse events and immune outcomes and collect preliminary data on respiratory symptoms to inform the design of a larger clinical trial.

### 2.2 Study population, recruitment, enrollment, and adherence

Study participants were long-stay elderly NH residents (≥65 y), recruited from NHs within approximately 1 h driving distance from the Boston area. Participants were recruited between May 2022 and June 2024. Interested volunteers capable of providing consent were asked to sign a screening consent form. The screening process was conducted by the study physician (AP).

A blood sample was obtained following screening consent if the volunteer met the inclusion criteria (see below). Participants with serum Zn levels (< 70 μg/dL) were enrolled and randomly assigned to one of the three groups. Group 1 served as the placebo-control and received a capsule containing ½ Recommended Dietary Allowance (RDA) of all the essential micronutrients (MN), including 5 mg of elemental Zn (½ RDA MN); Group 2 received a capsule containing ½ RDA MN including 30 mg/d elemental Zn, and Group 3 received a capsule containing ½ RDA MN including 60 mg/d elemental Zn. Zn was provided as Zn gluconate. The overall study design and number of subjects is shown in Figure 1. Both the study participants and researchers were blinded to group assignment throughout the intervention.

**Figure 1:**
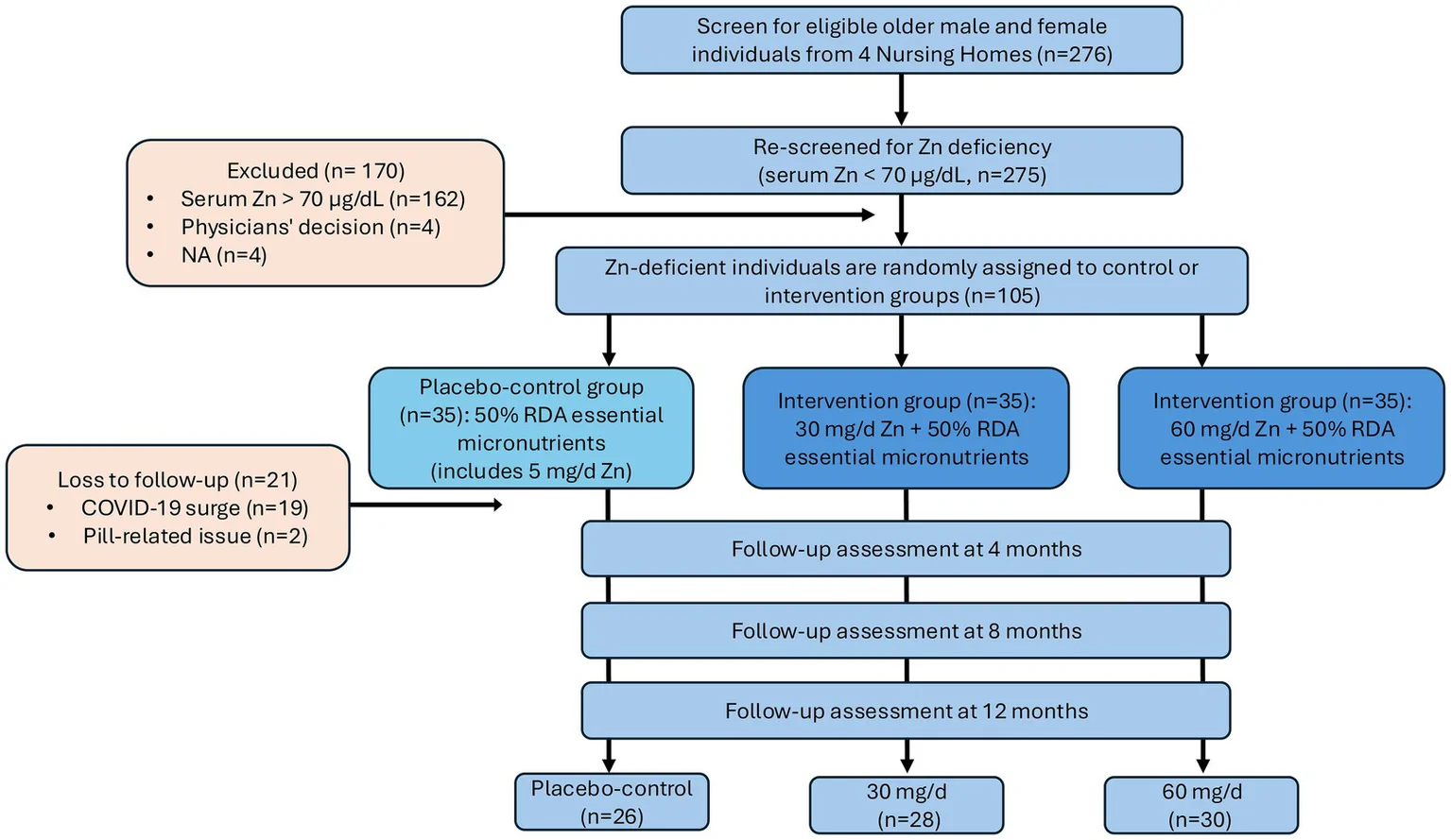
CONSORT flow diagram of participant enrollment, allocation, follow-up, and analysis. Of 276 individuals screened, 275 met the inclusion and exclusion criteria; 170 were excluded (162 zinc adequate, 4 physician decision, 4 other). A total of 105 zinc-deficient participants were randomized to placebo (½ RDA essential micronutrients; *n* = 35), 30 mg/d zinc (*n* = 35), or 60 mg/d zinc (*n* = 35), each with ½ RDA essential micronutrients. Between baseline and 4 months, 21 participants were lost to follow-up (19 due to COVID-19–related nursing home restrictions, 2 due to pill-related issues). Participants were followed at 4, 8, and 12 months. At 12 months, 26, 28, and 30 participants remained in the placebo, 30 mg/d, and 60 mg/d groups, respectively, and were included in the final analysis.

Adherence was directly monitored using a structured process integrated into routine nursing care. Monthly doses were pre-distributed and stored in the nursing home medication dispensing system. Nurses administered the capsules alongside routine morning medications, observing ingestion for residents requiring supervision. For independent residents, empty medication cups were collected and intake was verbally confirmed. Additionally, monthly pill counts were conducted by the study physician (AP) at the time each new batch was dispensed. While there remains a theoretical possibility that a resident may not ingest the medication despite an empty cup and verbal confirmation, adherence was considered effectively complete under these controlled conditions.

### 2.3 Vital signs and respiratory symptom reporting

Vital signs were obtained every 2 weeks. The parameters collected included temperature, blood pressure, heart rate, respiratory rate, and oxygen saturation. In addition, participants underwent a focused clinical examination assessing the lungs, heart, and sensation. Symptoms were also recorded during these visits, with particular attention to respiratory (cough, shortness of breath, sputum production, chest pain), gastrointestinal (nausea, vomiting, diarrhea, abdominal pain), and neurological complaints (headache, dizziness, altered sensation, weakness). Although symptom reports were sometimes corroborated with the nursing chart, this documentation was inconsistent, as participants may not always have reported symptoms to staff. Because symptoms could have resolved by the time of the examination, and due to reliance on self-report, there is potential for recall or reporting bias.

### 2.4 Inclusion and exclusion criteria

Participants were eligible if they were aged ≥ 65 years, had a > 6-month life expectancy as assessed by the study physician (AP), were willing to be randomized, were able to swallow pills, were not currently receiving antibiotics, were willing to receive the influenza vaccine, and had a BMI > 18 kg/m^2^ and albumin >3.0 g/dL. Participants currently taking RDA-level supplements were required to replace them with the study control supplement; calcium, vitamin D, and iron supplements were permitted. Participants continued their usual care throughout the study, with no restrictions on concomitant treatments beyond study-specific supplement protocols.

Exclusionary criteria included: anticipated transfer or discharge within 3-months of enrollment, bed- or room-bound continuous for the previous 3-months; presence of lung neoplastic diseases or other active neoplastic diseases requiring chemotherapy and/or immunosuppressive drugs (including ≤ 10 mg/day prednisone); nasogastric or tube-feeding; long-term (≥ 30 days) IV or urethral catheters; presence of tracheostomy or chronically ventilator-dependent; chronic prophylactic antibiotic treatment or long-term antibiotics; those with low albumin < 3.0 g/dL, indicative of inflammation/disease burden; a BMI < 18 kg/m^2^ due to significant underweight and nutrition risk; consumed supplements containing more than the RDA level of nutrients known to affect immune responses, i.e., vitamin E, C, B6, selenium, Zn, or β-carotene. Diagnosis of PNA or other infection at baseline did not exclude subject participation but did postpone enrollment to 4 wks after PNA symptoms had cleared.

### 2.5 Intervention

Participants received one of three daily capsules for 12 months: placebo (½ RDA of all essential micronutrients including 5 mg/day elemental Zn), 30 mg/day elemental Zn (½ RDA micronutrients plus additional Zn gluconate), or 60 mg/day elemental Zn (½ RDA micronutrients plus additional Zn gluconate). The complete micronutrient formulation, including chemical forms and exact daily doses for all three arms, is provided in Supplementary Table 1. The use of ½ RDA micronutrients as the placebo base was chosen because NH residents have heterogeneous multivitamin intake, and several micronutrients at sub-RDA levels have been shown to impair immune function. Providing ½ RDA as a base minimized variability due to background nutrient intake and was considered ethically appropriate given that participants were asked to discontinue their own supplements.

### 2.6 Primary endpoint(s)

The primary outcome of this study was to identify an effective and safe Zn supplementation dosage for NH residents. A dose was effective if it corrected Zn deficiency (>70 μg/dL), and safe if it was not associated with severe adverse events and did not adversely affect serum copper concentrations or other clinical chemistry parameters.

### 2.7 Secondary endpoints

#### 2.7.1 Adverse events and self-reported health changes

Participants were assessed at baseline and every two weeks for adverse events, categorized as follows: Minor side effects (nausea, bad taste) were documented but did not require discontinuation if participants chose to continue. Moderate side effects (nausea, vomiting, abdominal pain, diarrhea) were considered unacceptable and resulted in study withdrawal. Severe side effects (flu-like symptoms including fever, chills, and cough; neurological symptoms such as paresthesia, gait disturbance, or photophobia) also resulted in immediate withdrawal. If more than 10% of enrolled participants in any arm experienced at least one moderate or severe event, that arm would have been closed. Participants were also queried every 2 weeks regarding self-reported symptoms including cough, fever, and chills, which was tracked as a secondary outcome given its relevance as a respiratory symptom and its potential as a proxy signal for the PNA prevention trial to follow.

All capsules in this study contained 0.8 mg copper to mitigate the risk of Cu deficiency. In addition, the 60 mg/day elemental Zn dose used in the present study has been shown to be safe with respect to copper status. In a 6-week trial, 60 mg/day elemental Zn, as Zn gluconate, had no adverse effect on erythrocyte superoxide dismutase activity, a sensitive marker of copper depletion (17). Zn gluconate at lower doses (15–30 mg/day) has also been shown to have no adverse effects on folate, vitamin B12, or homocysteine status over 6 months in older adults (18), supporting the safety of Zn gluconate supplementation in the age range relevant to the present study.

### 2.8 Clinical chemistries

Blood was collected using appropriate Zn-free syringes, gloves, and tubes. Serum Zn concentration was measured by Quest Diagnostics (test code 945), a CLIA-certified and CAP-accredited reference laboratory, using inductively coupled plasma mass spectrometry (ICP-MS) with kinetic energy discrimination (KED) mode and helium as the collision cell gas to minimize polyatomic interferences (10). Internal standardization was used to correct for instrumental drift and matrix effects. Analytical performance met CLIA proficiency testing requirements, with imprecision < 20% CV at the limit of quantification and accuracy within ± 15% of target values. Quality control included certified reference materials analyzed at multiple concentration levels with each analytical run, and the laboratory participates in external proficiency testing programs (e.g., CAP surveys); inter-assay imprecision for Zn by ICP-MS in clinical laboratories is typically < 5–7% CV (19, 20). Serum albumin was measured by the bromcresol green (BCG) dye-binding colorimetric method, in which albumin binds BCG at acidic pH to form a colored complex measured spectrophotometrically (21, 22). Serum copper was measured by ICP-MS (10). Serum iron was measured by spectrophotometric methods consistent with ferrozine colorimetry, per International Committee for Standardization in Haematology (ICSH) recommendations (23, 24). Serum ferritin was measured by electrochemiluminescent immunoassay (ECLIA) (25). High-sensitivity CRP (hs-CRP) was measured by immunonephelometric assay, calibrated against WHO Reference Material 85/506 (26, 27). Complete blood count with differential (CBC-diff) was analyzed using a Baker 9,000 Hematology Analyzer using impedance and light-scatter detection to generate a five-part leukocyte differential, with blood smear confirmation of abnormal results (28, 29). All assays were performed under Quest Diagnostics validated standard operating procedures, with internal quality control and external proficiency testing (CAP surveys) in compliance with CLIA performance specifications.

### 2.9 Anthropometrics

BMI was calculated from baseline weight and standing or knee height (for subjects who cannot stand or are contracted) (30).

### 2.10 Frequencies of neutrophils and pan-T cells, and neutrophil-to-lymphocyte ratio (NLR)

Peripheral blood mononuclear cells for T-cell analysis were isolated from whole blood by density gradient centrifugation using SepMate™ PBMC Isolation Tubes (STEMCELL Technologies). Following isolation, cells were gated by forward and side scatter (FSC/SSC) to exclude debris and doublets, followed by staining with anti-human CD3-APC (clone REA613, Miltenyi Biotec) to identify pan-T cells (Supplementary Figure 1). For neutrophil analysis, whole blood was treated with eBioscience™ 1X RBC Lysis Buffer (Thermo Fisher Scientific), followed by FSC/SSC gating and staining with anti-human CD16b-APC antibody (clone REA589, Miltenyi Biotec) to identify neutrophils (Supplementary Figure 2). Isotype-matched controls were used during initial assay development to establish gating thresholds, which were then applied consistently across all study samples. Samples were analyzed using a BD Accuri C6 flow cytometer, and results are presented as the percentage of total leukocytes.

The NLR was calculated at each visit as absolute neutrophil count divided by absolute lymphocyte count, using the differential data already collected as part of routine clinical chemistry assessments.

### 2.11 Data management

Study data were collected using electronic Case Report Forms built in REDCap, a HIPAA- and 21 CFR 11-compliant platform hosted at the Human Nutrition Research Center on Aging at Tufts University. Forms were accessible via web browser or iPad application to accommodate variable internet connectivity at NH sites. Data were reviewed and adjudicated within 1 week of entry. Laboratory data were exported to Microsoft Excel, reviewed for outliers, and archived on secure servers with daily backups. Upon study completion, all data were de-identified and archived in standardized CSV format with documentation including variable naming conventions, value ranges, and data descriptions preserved for long-term storage and sharing.

### 2.12 Sample size estimation and statistical methods

Our plan to enroll 105 study participants (n = 35/group) was based on a power analysis of enrolling 27 study participants/group and allowing up to 20% attrition (loss of 7 participants/group) over the 1-year supplementation period as previously described in our study protocol (31). Data from our previous study demonstrated that 58% of subjects supplemented with 30 mg/day Zn exhibited conversion from Zn deficiency to sufficiency (11). We anticipated at least a 90% conversion for the 60 mg/d dose. To detect an increase in percent conversion from 58 to 90%, with 80% power and type I level of alpha = 0.05, 27 study participants per treatment group was required. Sample size was determined based on testing for a difference in percent conversion between the two dose groups using a one-sided Fisher’s Exact test.

Data were analyzed using an intent-to-treat approach to limit bias. Baseline differences in participant demographics and clinical chemistries were assessed using Chi-square tests for categorical variables and one-way ANOVA for continuous variables, with Tukey’s HSD post-hoc test for pairwise comparisons. Statistical significance was defined as *p* < 0.05. Changes in serum Zn concentrations across intervention groups over time were evaluated using mixed-effects linear regression models, with participant-ID included as a random effect to account for repeated measures. Missing data were handled using linear mixed-effects models, which use all available data under a missing at random assumption and do not require imputation. The proportion of participants achieving Zn adequacy was compared across groups using Chi-square tests. Due to incomplete longitudinal sampling and reduced samples size for the flow cytometry data, longitudinal mixed-effects modeling was not performed. Instead, cross-sectional comparisons were conducted at each available timepoint. Changes in clinical chemistries, NLR, and anthropometric measurements over time and by intervention arm were also assessed using mixed-effects linear regression models, with *p*-values adjusted for multiple comparisons using the Benjamini–Hochberg method, an adjusted *p*-value < 0.05 was considered significant.

## 3 Results

### 3.1 Participant demographics

As shown by Figure 1, of the 276 older adults screened, 275 met the inclusion and exclusion criteria. Of these, 105 met the serum Zn eligibility threshold (< 70 μg/dL) and were enrolled. The primary reason for ineligibility was serum Zn levels > 70 μg/dL, observed in approximately 58% of screened individuals. Notably, 38% of screened individuals had serum Zn values < 70 μg/dL – indicating a high prevalence of Zn deficiency in this population.

As shown in the CONSORT diagram (Figure 1), 84 participants successfully completed the study, with dropout numbers and reasons detailed therein. The primary reason for loss to follow-up during the intervention period was attributed to COVID-19 resurgence in the NHs. The remaining dropouts were due to pill-related issues: one participant found the pill size too large, and another disliked the taste, both in the 30 mg/d group. For participants who reported taste issues, we encouraged mixing the pills with food as a mitigation strategy.

As shown in Tables 1, 2, baseline demographics including age, BMI, sex, education, and race were well-balanced across groups with no statistically significant difference. Clinical chemistry parameters including serum Zn, albumin, iron, copper, CRP, potassium, and calcium were similarly balanced at baseline. No differences in complete blood count parameters, blood pressure, and red blood cell count were observed, confirming successful randomization.

**Table 1:** ZIPP study participants demographics.

Group	Control	30 mg/d	60 mg/d	*p*-value*
*n*	35	35	35	
Age (yrs) (± SD)	77.5 (± 9.0)	78.1 (± 8.6)	78.3 (± 9.8)	ns
BMI (kg/m^2^) (± SD)	23.9 (± 0.8)	24.0 (±0.8)	24.0 (± 0.8)	ns
Completed Highschool (*n*, %)	35 (100%)	35 (100%)	35 (100%)	ns
Sex (*n*, %)				ns
Female	15 (43%)	15 (43%)	15 (43%)	
Male	20 (57%)	20 (57%)	20 (57%)	
Gender (*n*, %)				ns
Female	15 (43%)	15 (43%)	14 (41%) ***	
Male	20 (57%)	20 (57%)	20 (57%)	
Race (*n*, %)				ns
Black	31 (88.6%)	29 (82.8%)	29 (85.3%)	
White	4 (11.4%)	6 (17.1%)	4 (11.8%)	
Asian	0 (0%)	0 (0%)	1 (2.9%)	
Ethnicity (*n*, %)				ns
Not Hispanic	32 (91.4%)	32 (91.4%)	33 (94.2%)	
Hispanic	3 (8.6%)	3 (8.6%)	2 (5.7%)	
Nursing home (*n*, %)				ns
Mattapan	19 (54.2%)	22 (62.8%)	25 (71.4%)	
Serenity Hill	4 (11.4%)	5 (14.2%)	4 (11.4%)	
West Roxbury	3 (8.5%)	3 (10.7%)	1 (3.3%)	
Edgar P Benjamin **	9 (25.7%)	5 (14.2%)	5 (14.2%)	

^***^Gender not reported.

^**^NH with COVID-19 surge—all participants were dropped before Visit 2.

^*^p-values are adjusted for multiple comparisons.

**Table 2:** ZIPP study participants clinical chemistries at baseline.

Group	Control	30 mg/d	60 mg/d	*p*-value^+^
*n*	35	35	35	
Zinc (μg/dL, ± SD)	57.2 (± 9.9)	55.4 (± 12.2)	56.3 (± 9.4)	ns
Albumin (g/dL, ± SD)	3.7 (± 0.3)	3.9 (± 0.4)	4.0 (± 0.4)	ns
Iron (μg/dL, ± SD)	98.9 (± 37.1)	86.3 (± 33.1)	88.5 (± 33.1)	ns
Copper (μg/dL, ± SD)	96.3 (± 25.2)	92.4 (± 22.8)	98.1 (± 24.6)	ns
TIBC (μg/dL, ± SD)	320.5 (± 54.6)	301.8 (± 44.3)	302.8 (± 48.0)	ns
Ferritin (ng/mL, ± SD)	117.0 (±116.6)	100.0 (±107.1)	114.0 (±122.7)	ns
CRP (mg/L, ± SD)	1.9 (± 1.1)	2.4 (± 2.6)	1.9 (± 1.0)	ns
WBC (± SD)	5.4 (± 1.6)	5.3 (± 1.6)	5.5 (± 1.9)	ns
Hemoglobin (g/dL, ± SD)	13.7 (± 0.8)	13.6 (± 0.6)	13.7 (± 0.5)	ns
Platelet (cells/μL, ± SD)	252.8 (± 68.0)	275.5 (± 63.0)	259.7 (± 74.0)	ns
Neutrophils (cells/μL, ± SD)	2854.3 (± 1548.9)	2696.9 (± 1504.7)	2577.7 (± 1174.1)	ns
Lymphocytes (cells/μL, ± SD)	1427.8 (± 788.2)	1434.4 (758.0)	1514.4 (± 852.1)	ns
Monocytes (cells/μL, ± SD)	457.3 (± 178.8)	413.0 (± 164.9)	447.3 (± 189.9)	ns
Eosinophils (cells/μL, ± SD)	150.0 (± 155.5)	142.7 (± 171.3)	123.3 (± 154.3)	ns
Basophils (cells/μL, ± SD)	76.0 (± 66.3)	71.1 (± 56.5)	69.9 (± 58.0)	ns
Glucose (mg/dL, ± SD)	77.7 (± 10.5)	80.8 (± 15.0)	80.0 (± 10.2)	ns
Urea nitrogen (mg/dL, ± SD)	15.2 (± 6.1)	18.0 (± 6.1)	17.7 (± 6.6)	ns
Hematocrit (%, ± SD)	41.4 (± 1.6)	40.9 (± 1.9)	40.4 (± 1.5)	ns
Mean corpuscular volume (fL, ± SD)	91.5 (± 5.3)	91.1 (± 5.2)	90.1 (± 4.6)	ns
MCH (pg, ± SD)	29.8 (± 1.7)	30.3 (± 1.7)	29.8 (± 1.6)	ns
MCHC (g/dL, ± SD)	33.8 (± 1.2)	33.7 (± 1.1)	33.7 (± 0.7)	ns
Creatinine (mg/dL, ± SD)	0.98 (± 0.15)	1.03 (± 0.14)	0.99 (± 0.18)	ns
Sodium (mmol/L, ± SD)	138.2 (± 3.6)	138.4 (± 4.0)	139.4 (± 2.8)	ns
Potassium (mmol/L, ± SD)	4.31 (± 0.5)	4.23 (± 0.5)	4.31 (± 0.5)	ns
Calcium (mg/dL, ± SD)	9.13 (± 0.5)	9.07 (± 0.5)	9.2 (± 0.5)	ns
Systolic pressure (mmHg, ± SD)	129.6 (± 5.8)	126.9 (± 5.7)	128.5 (± 4.3)	ns
Diastolic pressure (mmHg, ± SD)	75.2 (± 6.0)	74.2 (± 5.6)	74.2 (± 5.2)	ns

^+^*p*-values are adjusted for multiple comparisons. With the exception of serum zinc, all values shown fell within normal clinical reference ranges as reported by the performing laboratory (Quest Diagnostics) at the time of assay.

### 3.2 Primary endpoint(s)

#### 3.2.1 Serum Zn and clinical chemistries

The average baseline serum Zn levels were comparable across all three groups (Table 2). All other baseline clinical chemistries and WBC differentials were largely within normal ranges across groups (Table 2). To establish the optimal dose of Zn supplementation to achieve adequate serum Zn (≥ 70 μg/dL) in most participants (> 90%) within the shortest duration, we compared the proportion of participants reaching this threshold at each time point.

As shown in Figure 2 and Table 3, at 4 months, 4 (14.2%) and 8 (26.6%) participants in the 30 and 60 mg/d groups, respectively, reached the threshold (≥ 70 μg/dL), while no participants in the placebo group achieved Zn adequacy levels (4 month: 60 mg/d vs. placebo, *p* < 0.05). While the difference in proportions was statistically significant, 4 months was insufficient to convert > 90% of participants. At 8 months, 13 (46.4%) and 30 (100%) participants in the 30 and 60 mg/d groups, respectively, reached the threshold, while 0 participants in the placebo control reached adequate serum Zn levels. Both intervention group proportions were significantly different from the placebo control proportion (*p* < 0.001) by Fisher’s exact test. However, only the 60 mg/d group achieved the > 90% conversion threshold by 8 months. At 12 months, proportions remained unchanged from the 8-month timepoint in both intervention groups, with 13 of 28 (46.4%) in the 30 mg/day group and 30 of 30 (100%) in the 60 mg/day group sustaining Zn adequacy.

**Figure 2:**
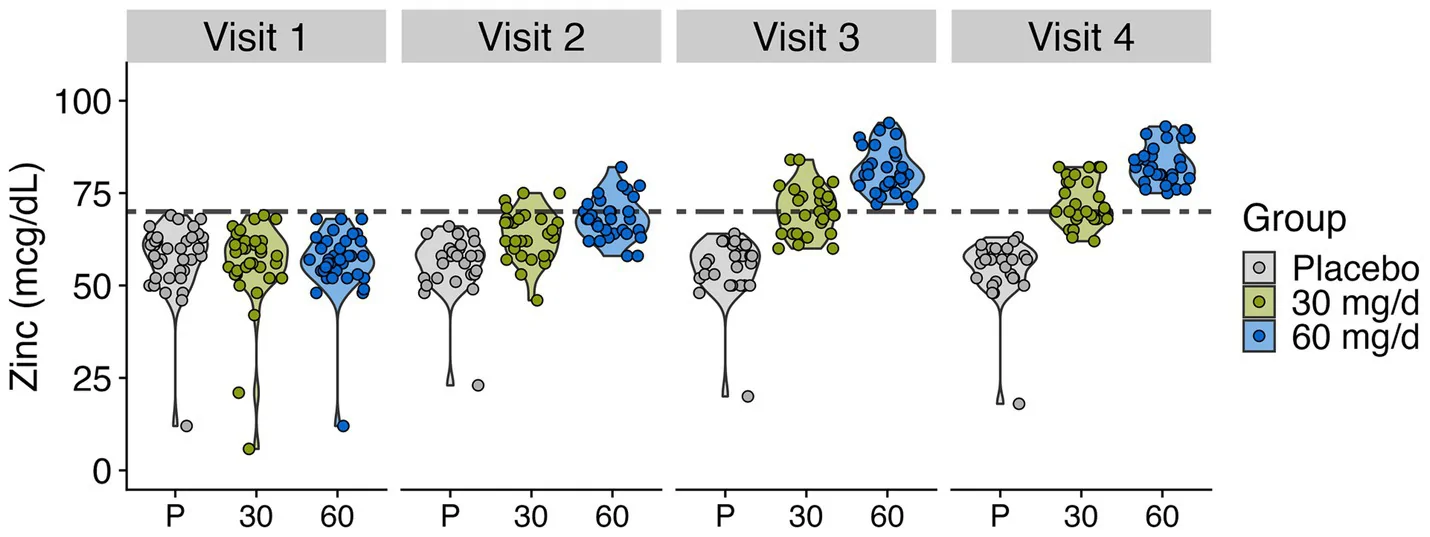
Serum zinc concentrations over time by supplementation group. Serum zinc (mcg/dL) levels were measured at baseline (Visit 1), 4 (Visit 2), 8 (Visit 3), and 12 months (Visit 4) in participants receiving placebo, 30 mg/d zinc, or 60 mg/d zinc. Each dot represents an individual participant. *n* = 26–35/group. Statistical comparisons are reported in Tables 3, 7.

**Table 3:** Number of participants above or below the threshold (> 70 μg/dL).

Zn status(>70 μg/dL)	Baseline			4 months			8 months			12 months		
>70 mcg/dL	P^a^	30^a^	60^a^	P^a^	30^a,b^	60^b^	P^a^	30^b^	60^c^	P^a^	30^b^	60^c^
No	35	35	35	26	24	22	26	15	0	26	15	0
Yes	0	0	0	0	4	8	0	13	**30**	0	13	**30**

^*^Values represent the number of participants above (>70 μg/dL) or below the threshold at each timepoint. Within each timepoint, groups that do not share a common letter are significantly different (Fisher’s exact test, p < 0.05, Benjamini-Hochberg adjusted p < 0.05, corrected across all pairwise comparisons and timepoints). Differences in n between baseline and 4 months are due to dropouts.

P = placebo; 30 = 30 mg/day; 60 = 60 mg/day.

To examine whether potential covariates influenced serum Zn concentrations, mixed-effects linear models were fitted including race, sex, BMI, CRP, and albumin as predictors — the latter two given their known transient effects on serum Zn (32), and the former three included as pre-specified demographic covariates. None of these variables were significantly associated with serum Zn, and unadjusted estimates were therefore reported.

### 3.3 Frequences of neutrophils and pan-T cells

Longitudinal trajectories of pan-T cells were higher in the 60 mg/d Zn supplemented groups compared to placebo (Supplementary Figure 3). Linear mixed-effects modeling revealed a trend toward a group-by-time interaction, with the 60 mg/d group demonstrating an increasing trajectory relative to placebo (β = 5.57, *p* = 0.072). Mean pan-T cell percentage in the 60 mg/day group increased from 23.6% (± 13.1) at baseline to 37.8% (± 18.1) at 12 months, compared to a decrease from 21.7% (± 11.2) to 16.5% (± 17.6) in placebo and a modest increase from 18.7% (± 14.1) to 20.2% (± 5.56) in the 30 mg/day group; sample sizes at the 12-month timepoint were small (n = 4–6 per group) due to attrition and limited sample availability for flow cytometry.

The NLR showed no significant main effect of time (*p* = 0.38) or Time×Group interaction for the 30 mg/day (*p* = 0.55) or 60 mg/day (*p* = 0.22) groups relative to placebo over the 12-month intervention period (Supplementary Table 2).

### 3.4 Impact of zinc on micronutrients and biomarkers

As a secondary analysis, we assessed whether clinical chemistries and anthropometric measures changed differently over time according to group assignment.

As shown in Tables 4, 5, mixed effects modeling indicated a significant relative decrease in BMI in both the 30 mg/day (−0.3 kg/m^2^, *p* < 0.001) and 60 mg/day (−0.7 kg/m^2^, *p* < 0.001) groups, while increasing in the placebo group (+0.7 kg/m^2^) over the intervention period (Figure 3). A modest but significant effect on hematocrit was observed in the 60 mg/day group relative to placebo (*β* = 0.111, *p* < 0.05), but not in the 30 mg/day group (β = 0.044, ns), suggesting a dose-dependent trend. No significant changes were observed in hemoglobin, mean corpuscular hemoglobin (MCH), mean corpuscular hemoglobin concentration (MCHC), mean corpuscular volume (MCV), white blood cell count, platelet count, or differential counts (basophils, eosinophils, lymphocytes, monocytes, neutrophils), nor in serum creatinine across intervention groups (Tables 4, 5).

**Table 4:** Anthropometric and clinical chemistries of ZIPP study participants over time by supplementation group.

Metric	Baseline			4 months			8 months			12 months		
	Placebo	30 mg/d	60 mg/d	Placebo	30 mg/d	60 mg/d	Placebo	30 mg/d	60 mg/d	Placebo	30 mg/d	60 mg/d
N	35	35	35	26	28	30	26	28	30	26	28	30
BMI (kg/m^2^; ±SD)	23.9 (0.9)	24.0 (0.8)	24.1 (0.8)	24.0 (0.8)	24.0 (0.8)	23.9 (0.8)	24.4 (0.7)	23.9 (0.6)	23.7 (0.7)	24.6 (0.8)	23.7 (0.5)	23.4 (0.7)
Albumin (g/dL; ±SD)	3.7 (0.3)	4.0 (0.4)	4.0 (0.5)	3.9 (0.3)	4.1 (0.4)	4.1 (0.4)	4 (0.3)	4.2 (0.4)	4.2 (0.3)	4.0 (0.3)	4.3 (0.4)	4.4 (0.3)
CRP (mg/L; ±SD)	1.9 (1.1)	2.5 (2.7)	1.9 (1.0)	1.5 (0.9)	1.5 (0.8)	1.5 (0.8)	2.0 (1.4)	2.3 (0.6)	2.1 (1.5)	2.0 (1.4)	2.2 (1.5)	1.9 (1.0)
Basophils (cells/μL, ±SD)	76.0 (66.3)	71.1 (56.5)	70.0 (58.0)	65.9 (59.3)	73.6 (48.9)	88.5 (55.4)	60.8 (46.6)	78.9 (57.9)	90.4 (62.2)	45.3 (44.2)	63.3 (47.6)	69.6 (60.4)
Eosinophils (cells/μL, ±SD)	150.0 (155)	142.8 (171.3)	123.3 (154.3)	141.3 (137.5)	136.3 (148.0)	139.2 (138.4)	218.1 (161.9)	231.3 (160.8)	302.4 (131.1)	217.6 (166.7)	155.1 (151.5)	172.8 (161.6)
Lymphocytes (cells/μL, ±SD)	1426.8 (787.2)	1434.5 (758.0)	1514.5 (852.2)	1807.4 (911.6)	1658.7 (831.1)	1510.7 (714.4)	1849.3 (888.1)	1918.2 (876.4)	2207.8 (853.2)	1845.0 (753.3)	1852.6 (788.7)	2275.6 (886.7)
Monocytes (cells/μL, ±SD)	457.3 (178.8)	413.0 (165.0)	447.4 (189.9)	471.5 (158.6)	478.4 (180.5)	553.7 (174.7)	564.8 (149.5)	680.2 (198.9)	617.6 (175.3)	550.3 (212.4)	556.6 (205.7)	625.2 (184.0)
Neutrophils (cells/μL, ±SD)	2854.3 (1548.9)	2697.0 (1504.7)	2577.7 (1174.1)	3023.2 (1512.1)	2562.8 (1117.0)	2911.0 (1303.9)	3018.7 (1491.3)	3232.8 (1592.7)	3469.2 (1619.2)	3451.4 (1785.3)	3267.5 (1510.2)	3003.2 (1278.1)
Creatinine (mg/dL, ±SD)	0.9 (0.1)	1.0 (0.2)	1.0 (0.2)	0.9 (0.1)	1.0 (0.1)	1.0 (0.1)	1.0 (0.1)	1.0 (0.1)	1.1 (0.1)	1.1 (0.1)	1.0 (0.1)	1.0 (0.1)
Diastolic (mmHg, ±SD)	75.2 (6.0)	74.3 (5.6)	74.2 (5.3)	75.6 (4.9)	75.1 (4.2)	76.0 (4.0)	75.3 (4.9)	73.1 (4.8)	74.1 (5.6)	73.3 (5.4)	73.1 (5.8)	73.7 (4.8)
Systolic (mmHg, ±SD)	129.6 (5.8)	126.9 (5.8)	128.5 (4.3)	128.9 (5.0)	131.3 (8.5)	128.5 (5.5)	129.9 (7.3)	128.4 (6.2)	128.7 (5.6)	131.2 (9.2)	130.5 (9.0)	128.9 (8.9)
Ferritin (ng/mL, ±SD)	117.0 (116.6)	100.0 (107.1)	114.5 (122.8)	103.2 (104.9)	112.5 (114.5)	114.4 (117.4)	165.0 (120.7)	158.0 (130.5)	164.4 (126.1)	113.8 (103.8)	161.5 (119.3)	145.1 (109.3)
Glucose (mg/dL, ±SD)	77.8 (10.5)	80.8 (15.1)	80.1 (10.3)	82.8 (9.8)	80.2 (7.3)	84.3 (12.6)	81.4 (8.3)	85.8 (20.7)	84.1 (9.7)	84.0 (9.7)	81.1 (9.6)	84.1 (8.1)
Hematocrit (%, ±SD)	41.4 (1.6)	40.9 (1.9)	40.4 (1.5)	41.0 (1.9)	41.4 (1.6)	41.0 (2.0)	41.9 (1.4)	42.0 (1.7)	42.2 (1.7)	42.1 (1.9)	42.2 (1.7)	42.5 (1.4)
Hemoglobin (g/dL, ±SD)	13.7 (0.8)	13.6 (0.6)	13.7 (0.6)	13.7 (0.7)	13.9 (0.7)	13.8 (0.7)	13.9 (0.5)	14.0 (0.4)	14.1 (0.4)	14.0 (0.6)	13.8 (0.4)	14.0 (0.5)
MCH (pg, ±SD)	29.8 (1.7)	30.4 (1.8)	29.9 (1.6)	30.6 (1.5)	31.0 (1.5)	30.6 (1.7)	31.2 (1.5)	31.2 (1.2)	31.3 (1.5)	31.3 (1.5)	31.3 (1.3)	31.2 (1.6)
MCHC (g/dL, ±SD)	33.8 (5.3)	33.7 (1.1)	33.8 (0.8)	33.6 (1.0)	33.7 (1.0)	33.9 (1.0)	34.1 (0.6)	34.1 (0.7)	34.1 (0.7)	34.5 (0.6)	34.3 (0.7)	34.4 (0.6)
MCV (fL, ±SD)	91.5 (5.3)	91.1 (5.2)	90.2 (4.6)	90.6 (4.6)	92.8 (4.8)	92.2 (4.0)	91.8 (4.4)	89.7 (4.1)	92.8 (3.5)	89.5 (5.3)	91.8 (5.6)	90.2 (4.9)
Platelets (±SD)	252.8 (68.0)	275.6 (63.0)	259.7 (74.0)	296.6 (74.6)	289.1 (72.2)	275.3 (65.8)	309.4 (57.0)	319.8 (63.3)	324.1 (60.7)	327.2 (54.5)	307.9 (55.8)	320.3 (55.8)
TIBC (μg/dL, ±SD)	320.5 (54.6)	301.9 (44.3)	302.9 (48.0)	320.5 (54.6)	322.0 (48.2)	306.3 (51.1)	326.4 (40.3)	323.3 (50.4)	324.6 (43.3)	315.1 (47.1)	316.8 (37.9)	339.3 (48.0)
WBC (±SD)	5.4 (1.6)	5.3 (1.6)	5.5 (2.0)	5.5 (1.7)	5.6 (1.8)	5.1 (1.6)	5.9 (1.8)	6.7 (2.5)	6.8 (2.6)	7.0 (2.5)	6.3 (2.3)	6.2 (2.0)

Statistical comparisons were performed using linear mixed-effects models; results are summarized in Table 5.

**Table 5:** Linear mixed-effects model estimated coefficients of clinical chemistries by supplementation group over time.

Metric	Comparison	Estimate (β)	Standard Error	*p*-value*
BMI (kg/m^2^)	Placebo vs. 30	−0.084	0.008	<0.001
	Placebo vs. 60	−0.106	0.007	<0.001
Albumin (g/dL)	Placebo vs. 30	0.010	0.006	ns
	Placebo vs. 60	0.011	0.006	ns
CRP (mg/L)	Placebo vs. 30	−0.013	0.034	ns
	Placebo vs. 60	−0.012	0.034	ns
Diastolic (mmHg)	Placebo vs. 30	0.005	0.143	ns
	Placebo vs. 60	0.065	0.142	ns
Systolic (mmHg)	Placebo vs. 30	0.088	0.196	ns
	Placebo vs. 60	−0.095	0.194	ns
Glucose (mg/dL)	Placebo vs. 30	−0.294	0.339	ns
	Placebo vs. 60	−0.142	0.332	ns
Hematocrit (%)	Placebo vs. 30	0.044	0.0419	ns
	Placebo vs. 60	0.111	0.0414	<0.05
Hemoglobin (g/dL)	Placebo vs. 30	−0.002	0.0156	ns
	Placebo vs. 60	0.016	0.0154	ns
MCH (pg)	Placebo vs. 30	−0.051	0.0427	ns
	Placebo vs. 60	−0.012	0.0423	ns
MCHC (g/dL)	Placebo vs. 30	−0.006	0.0236	ns
	Placebo vs. 60	−0.002	0.0235	ns
MCV (fL)	Placebo vs. 30	0.095	0.1345	ns
	Placebo vs. 60	0.152	0.1332	ns
WBC	Placebo vs. 30	−0.0175	0.055	ns
	Placebo vs. 60	−0.022	0.0547	ns
Platelets	Placebo vs. 30	−2.71	1.751	ns
	Placebo vs. 60	−0.22	1.733	ns
Basophils (cells/μL)	Placebo vs. 30	2.00	1.613	ns
	Placebo vs. 60	2.55	1.592	ns
Eosinophils (cells/μL)	Placebo vs. 30	−3.37	4.457	ns
	Placebo vs. 60	1.29	4.410	ns
Lymphocytes (cells/μL)	Placebo vs. 30	4.1	23.6	ns
	Placebo vs. 60	38.9	23.46	ns
Monocytes (cells/μL)	Placebo vs. 30	6.96	5.307	ns
	Placebo vs. 60	5.92	5.275	ns
Neutrophils (cells/μL)	Placebo vs. 30	14.47	41.511	ns
	Placebo vs. 60	4.50	41.416	ns
Creatinine (mg/dL)	Placebo vs. 30	−0.008	0.004	ns
	Placebo vs. 60	−0.005	0.004	ns

^*^P-values are adjusted for multiple comparisons.

Estimates are derived from linear mixed-effects models including fixed effects for group, time (coded in months: 0, 4, 8, 12), and their interaction, with a random intercept for participant. β represents the Group × Time interaction coefficient, reflecting the estimated difference in the monthly rate of change for each metric relative to placebo.

**Figure 3:**
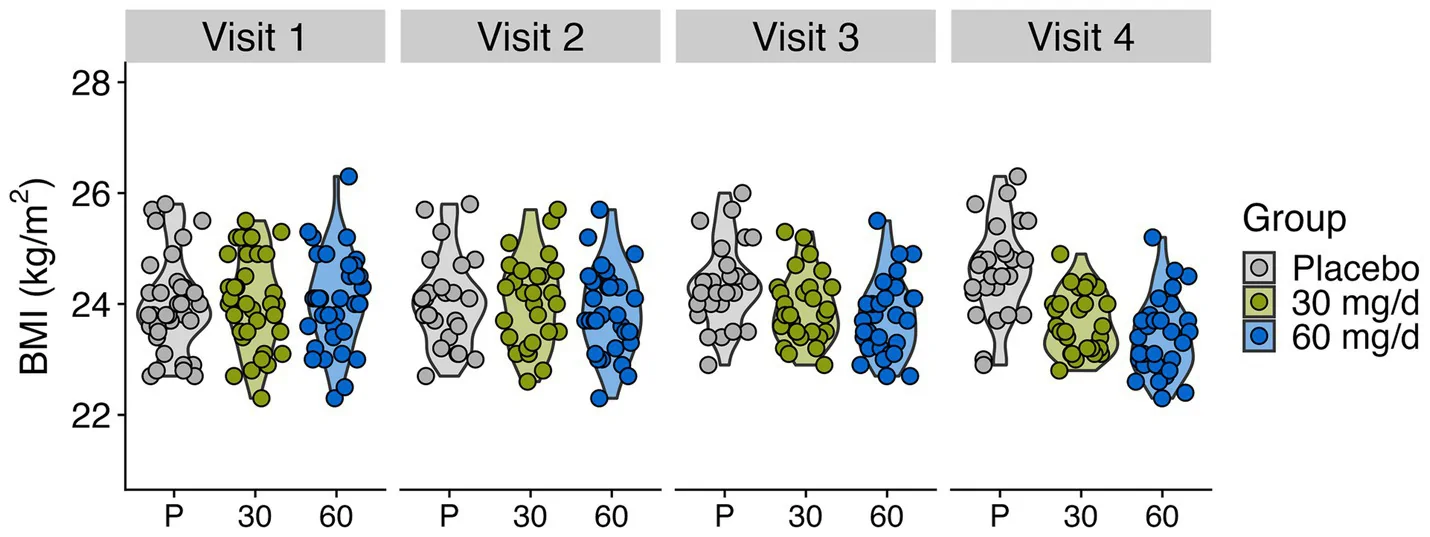
Distribution of body mass index (BMI) over time by supplementation group. Violin plots show BMI (kg/m2) for each participant at baseline (Visit 1), 4 (Visit 2), 8 (Visit 3), and 12 months (Visit 4) in the placebo, 30 mg/d, and 60 mg/d zinc groups. Each dot represents an individual participant. n = 26–35/group. Statistical analyses are provided in Table 5.

Copper status, and micronutrient status for calcium, iron, potassium were unaffected by either supplementation dose (Tables 6, 7); changes in serum Zn did not correlate with changes in serum copper (Spearman ρ = 0.09, *p* = 0.39, Figure 4). No correlations were observed between changes in serum Zn and changes in other measured micronutrients including iron, calcium, or potassium throughout the intervention (Table 8). A modest but significant increase in total iron binding capacity (TIBC) was observed with the 60 mg/day dose (*p* < 0.05), potentially related to the observed increase in hematocrit in the 60 mg/d group.

**Figure 4:**
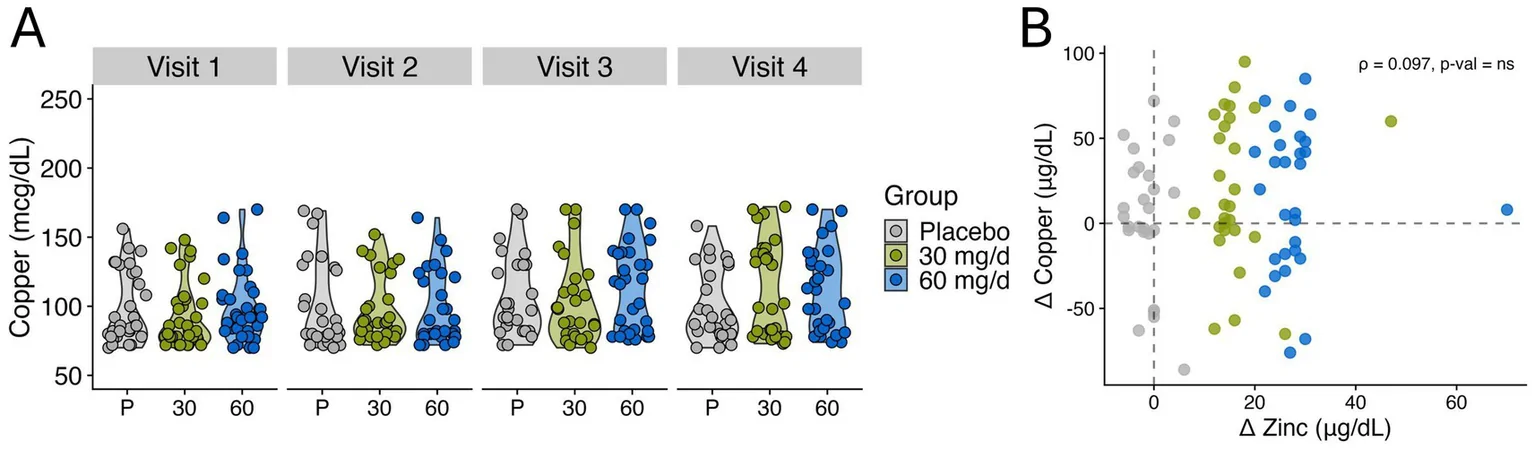
Serum copper, and copper–zinc relationship. **(A)** Serum copper (mcg/dL) levels were measured at baseline (Visit 1), 4 (Visit 2), 8 (Visit 3), and 12 months (Visit 4) in participants receiving placebo, 30 mg/d zinc, or 60 mg/d zinc. Each dot represents an individual participant. n = 26–35/group. Statistical comparisons are reported in Table 7. **(B)** Scatter plot showing the relationship between serum copper and zinc concentrations across all participants and timepoints. No correlation was observed (Spearman’s ρ and *p*-value).

**Table 6:** Micronutrient levels of ZIPP study participants over time by supplementation group.

Micronutrient	Baseline			4 months			8 months			12 months		
	Placebo	30 mg/d	60 mg/d	Placebo	30 mg/d	60 mg/d	Placebo	30 mg/d	60 mg/d	Placebo	30 mg/d	60 mg/d
N	35	35	35	26	28	30	26	28	30	26	28	30
Zinc (μg/dL)	57.2 (9.9)	55.4 (12.2)	56.3 (9.4)	55.6 (8.3)	63.5 (6.7)	68.3 (5.7)	54.7 (8.4)	70.3 (6.6)	81.4 (6.1)	54.2 (8.5)	72.4 (6.1)	83.1 (5.4)
Iron (μg/dL)	98.0 (37.1)	86.4 (33.1)	88.8 (33.2)	95.4 (38.1)	95.6 (37.5)	95.3 (32.8)	100.5 (30.5)	112.8 (38.1)	102.6 (35.3)	108.6 (36.1)	111.3 (41.1)	107.9 (36.3)
Calcium (mg/dL)	9.1 (0.5)	9.1 (0.5)	9.2 (0.5)	9.2 (0.4)	9.3 (0.6)	9.2 (0.5)	9.2 (0.5)	9.2 (0.4)	9.2 (0.5)	9.1 (0.6)	9.2 (0.5)	9.5 (0.6)
Copper (μg/dL)	96.3 (25.2)	92.4 (22.9)	98.2 (24.7)	101.3 (31.8)	97.7 (23.6)	97.2 (26.5)	107.1 (28.8)	102.8 (29.7)	112.8 (31.0)	101.0 (26.0)	112.8 (34.7)	111.8 (29.9)
Potassium (mmol/L)	4.4 (0.5)	4.3 (0.5)	4.3 (0.5)	4.4 (0.6)	4.3 (0.5)	4.5 (0.5)	4.4 (0.5)	4.5 (0.6)	4.3 (0.5)	4.2 (0.5)	4.3 (0.5)	4.3 (0.6)

Statistical comparisons were performed using linear mixed-effects models; results are summarized in Table 7.

**Table 7:** Linear mixed-effects model estimates of micronutrients of interest by supplementation group over time.

Metric	Comparison	Estimate (β)	Standard error	*p*-value*
Iron (μg/dL)	Placebo vs. 30	1.37	0.904	ns
	Placebo vs. 60	0.087	0.892	ns
Ferritin (ng/mL)	Placebo vs. 30	3.73	2.757	ns
	Placebo vs. 60	1.61	2.723	ns
TIBC (μg/dL)^+^	Placebo vs. 30	1.43	1.197	ns
	Placebo vs. 60	3.44	1.184	<0.05
Calcium (mg/dL)	Placebo vs. 30	0.008	0.0144	ns
	Placebo vs. 60	0.017	0.0143	ns
Potassium (mmol/L)	Placebo vs. 30	0.010	0.0153	ns
	Placebo vs. 60	−0.001	0.0152	ns
Copper (μg/dL)	Placebo vs. 30	1.109	0.764	ns
	Placebo vs. 60	0.874	0.760	ns
Zinc (μg/dL)	Placebo vs. 30	1.559	0.121	<0.0001
	Placebo vs. 60	2.563	0.120	<0.0001

^*^P-values are adjusted for multiple comparisons.

^+^TIBC = total iron binding capacity.

Estimates are derived from linear mixed-effects models including fixed effects for group, time (coded in months: 0, 4, 8, 12), and their interaction, with a random intercept for participant. β represents the Group×Time interaction coefficient, reflecting the estimated difference in the monthly rate of change for each metric relative to placebo.

### 3.5 Adverse events and self-reported symptoms

No moderate or severe adverse events were reported by any participants. As mentioned earlier, minor tolerability issues were infrequent and limited to two participants in the 30 mg/d group (pill size or taste), as described in the participant flow section, and are discussed further in the Discussion.

As a secondary, exploratory outcome, self-reported respiratory symptoms were collected at each of the 27 assessment timepoints across fourteen symptom categories, including cough, fever, chills, headache, muscle aches, night sweats, nausea, diarrhea, bloating, epigastric pain, constipation, metallic taste, tingling, and paresthesia; these data reflect participant- or caregiver-reported symptoms rather than clinician confirmed diagnoses. As shown in Figure 5A, coughs were reported significantly more often in the placebo group compared to both the 30 mg/day or 60 mg/day groups at 9 of the 27 timepoints (adjusted *p* < 0.05 at each); in all 9 cases, participants in the placebo group reported coughs more frequently than either supplemented group (Figure 5B). A similar, though less pronounced, pattern was observed for fever, which was significantly more frequent in the placebo group for 5 of 27 timepoints (Figure 5A, adjusted *p* < 0.05). Chills were reported at a higher frequency in the placebo group for 1 of the 27 instances (Figure 5A, adjusted *p* < 0.05). The co-occurrence of elevated cough, fever, and chills in the placebo group across multiple timepoints suggests a consistent pattern of greater respiratory symptom burden in un-supplemented participants. All self-reported symptom counts and analyses are provided as a Supplementary file.

**Figure 5:**
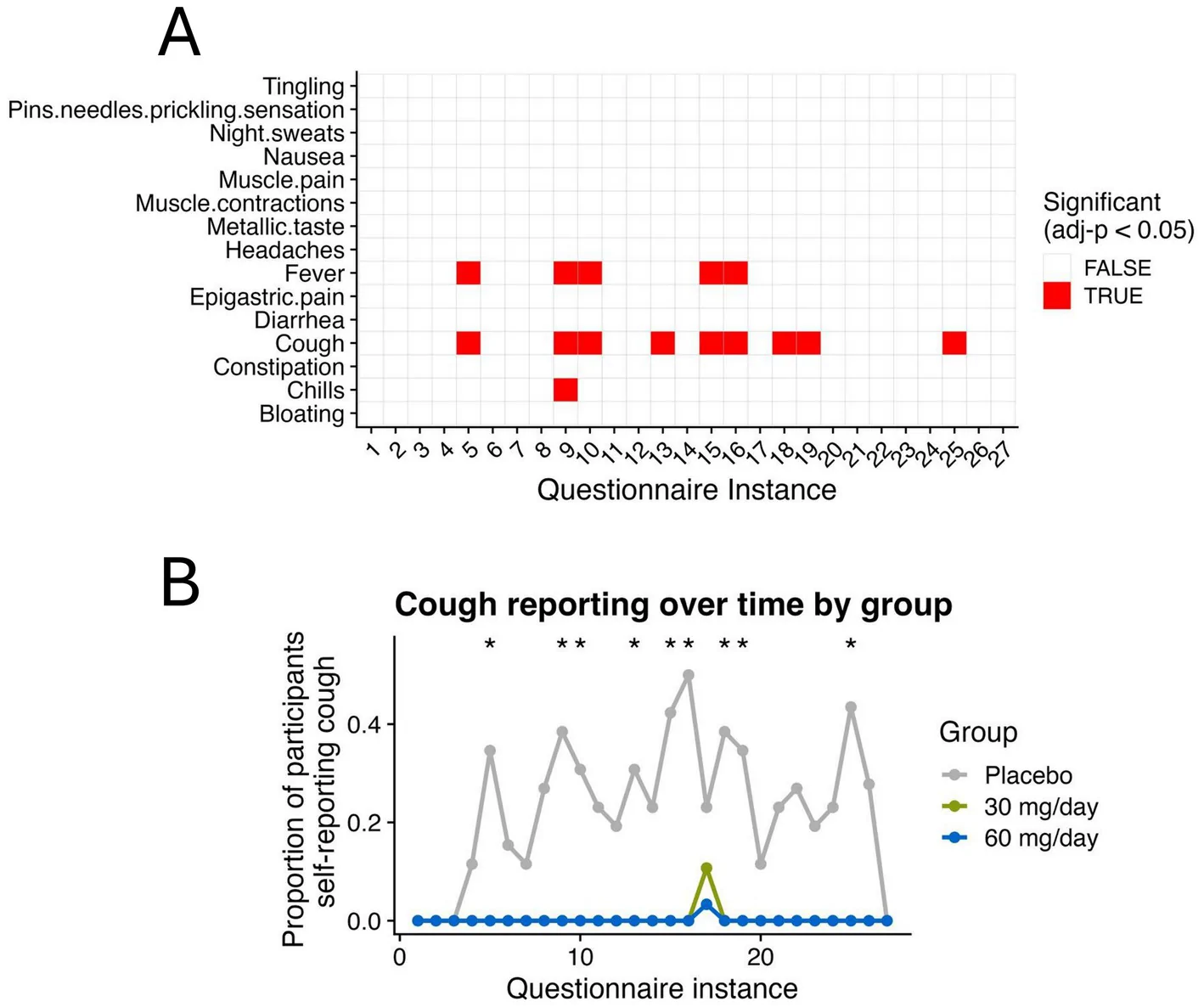
Longitudinal self-reported symptoms by supplementation group over time. **(A)** Heatmap showing the occurrence of self-reported symptoms across ~27 questionnaire instances (baseline and every 2 weeks) in participants assigned to placebo, 30 mg/day, or 60 mg/day zinc. Each tile indicates whether the difference between groups for that symptom and instance was statistically significant (adjusted *p* < 0.05, red) or not significant (white). Cough, fever, and chills were more frequently reported in the placebo group. **(B)** Line plot showing the proportion of participants reporting cough over time by group. Each point represents the proportion of participants responding “Yes” at a given questionnaire instance. Asterisks (*) indicate instances where the placebo group reported significantly more cough events than both the 30 mg/d and 60 mg/day groups (Fisher’s exact test, Holm-adjusted *p* < 0.05).

## 4 Discussion

### 4.1 Main findings and dose efficacy

This randomized placebo-controlled and double-blind clinical study demonstrates that supplementation with 30 or 60 mg/d Zn effectively corrects serum Zn levels in Zn-deficient older adults residing in senior NHs without adverse effects. These findings build on our earlier observational study and clinical trial, in which NH residents received 30 mg/d elemental Zn for 3 months (13), and helps address an important gap in identifying the optimal dose needed to restore Zn levels in this population, a key prerequisite for any clinical trial aimed at determining efficacy of Zn against respiratory diseases. The 60 mg/day dose achieved Zn adequacy in 100% of participants by month 8, while 30 mg/day restored adequacy in only 46.4% of participants after 12 months, consistent with our prior finding that a subset of severely Zn-deficient older adults may require higher than 30 mg/d of elemental Zn to reach the target threshold. Longer periods of supplementation did not improve serum Zn levels beyond that observed at 8 months in the 30 mg/d elemental Zn group.

### 4.2 Population characteristics and anthropometric changes

Interestingly, the proportion of Zn-deficient individuals in this study (38%) falls within the range of the previously reported NH population from our group (~30–58%) (11, 13). In the current study, ~85% of participants were Black, compared to the predominantly White populations in our earlier studies (11, 13). While racial and socioeconomic disparities in micronutrient status may contribute to variability in Zn deficiency prevalence, additional factors are more likely contribute to differences in prevalence. This is supported by the observation that the prevalence of Zn deficiency in our previous two studies, with predominantly White populations, ranged from 30 to 58%, compared to that observed in the current study. Notably, the three studies were conducted over a decade apart, and secular changes in nutritional status, clinical care, or population characteristics, e.g., sex, BMI, etc., may have influenced baseline Zn levels. We also observed a modest but statistically significant decrease in BMI in both Zn-supplemented groups relative to placebo over the 12-month intervention period. Mean BMI decreased from 24.1 to 23.4 kg/m^2^ (−0.7 kg/m^2^, approximately 2.9%) in the 60 mg/day group and from 24.0 to 23.7 kg/m^2^ (−0.3 kg/m^2^, approximately 1.3%) in the 30 mg/day group, while BMI in the placebo group increased slightly from 23.9 to 24.6 kg/m^2^ (+0.7 kg/ m^2^). Clinically significant weight loss in nursing home populations is generally defined as a decline exceeding 5–7% within 90 days, the magnitude and pace of BMI decline observed here falls well below this threshold, and all values remained within the normal range throughout the study (23–25 kg/m^2^). However, BMI does not distinguish between loss of lean vs. fat mass, and unintentional lean mass loss is a recognized risk factor for frailty and adverse outcomes in older adults independent of absolute weight change. As body composition was not assessed in this study, we cannot determine whether the observed BMI decline reflects fat mass, lean mass or a combination of both. Further, while we did not observe any associated adverse clinical indicators, this finding warrants closer scrutiny, and we recommend that body composition be assessed in future trials.

### 4.3 Zn intervention on nutrient status, baseline characteristics, and immune cells

Zn supplementation concurrent with half-RDA micronutrient supplementation did not adversely affect copper status, as evidenced by no significant change in serum copper levels across the intervention period. Correlation analysis of changes in serum Zn with changes in serum copper showed no negative correlation; indeed, the overall trend was weakly positive, possibly reflecting a broader improvement in nutritional status among supplemented participants rather than any Zn-copper antagonism at these doses. However, it is important to note that the 60 mg/day elemental Zn dose used in this study exceeds the UL of 40 mg/day established by IOM for Zn (15). The UL was derived from studies of replete individuals and was intended to define a threshold below which adverse effects would be unlikely to occur, principally copper deficiency, in the general population. However, it was not designed to define a safe ceiling for supplementation in Zn-deficient individuals. Our findings, showing no adverse effect on serum copper at the high Zn dose over 12 months, suggests that exceeding the UL in a Zn-deficient NH population under monitored conditions may be feasible and safe; however, this study did not directly test whether the UL itself should be revised for this population. Further, we assessed copper status using total serum copper, which does not exclude potential effects on other markers of copper status, such as ceruloplasmin or erythrocyte copper/Zn-superoxide dismutase activity, which may be more sensitive to early or subclinical copper depletion. In parallel, our findings are derived from a 12-month supplementation period and thus, we cannot rule out copper deficiency risk with continued Zn supplementation beyond this period; longer-term surveillance would be needed to establish safety with extended use.

The absence of significant associations between baseline participant characteristics — including sex, race, BMI, CRP, and albumin – and serum Zn response suggests that the intervention effect was robust across participant subgroups, supporting the generalizability of the findings within this population.

The NLR, a recognized marker of immune and nutritional status in geriatric populations, was calculated from absolute neutrophil and lymphocyte counts collected at each visit. Mixed-effects modeling showed no significant effect of Zn supplementation on NLR at either dose over the 12-month intervention period.

Although not statistically significant, we observed an increase in the proportion of circulating pan-T cells at later timepoints in the high dosage Zn supplemented groups. This directional increase is consistent with our own prior studies and those by others which demonstrate that Zn supplementation supports T-cell homeostasis and function (12, 13, 33–35). We note important limitations to our immune cell phenotyping: T cells (CD3+) and neutrophils (CD16b+) were identified using FSC/SSC gating alone; CD16b is also not fully specific to neutrophils (it is expressed on basophils and eosinophils, and other granulocyte populations) and is shed during activation, and CD3 does not exclude small number of CD3 expressing NKT-like cells. Additionally, because frequencies are assessed as a proportion, changes in this metric can be influenced by shifts in other leukocyte populations rather than reflecting a true change in absolute T-cell abundance. Given this limitation, the marginal statistical trend observed (*p* = 0.072), and reduced sample availability, these findings should be interpreted as exploratory rather than confirmatory. Future trials should employ a more rigorous panel (e.g., CD45 backbone, viability dye, CD66b/CD15 for neutrophils). A larger clinical trial should also be adequately powered for immune phenotyping and should include measurement of both intracellular total and labile Zn pools.

### 4.4 Future implications

Our findings have important implications for designing future multi-site clinical trials of Zn supplementation to prevent respiratory infections. Based on these results, a dose of 60 mg/day would be effective in restoring serum Zn levels in this population and could be used to evaluate whether Zn supplementation can reduce the risk of infections such as PNA.

**Table 8:** Micronutrient correlations.

Metric	ρ	*p*-value*
Δ Zinc x Δ Copper	0.09	ns
Δ Zinc x Δ Iron	0.14	ns
Δ Zinc x Δ Calcium	0.14	ns
Δ Zinc x Δ Potassium	0.05	ns

^*^Spearman correlation using pair-wise comparisons, a p-value < 0.05 was considered significant.

### 4.5 Strengths and limitations

Collectively, this study demonstrates that: (a) a significant proportion of otherwise healthy older adults residing in NH have low serum Zn levels; (b) a safe and effective dose (60 mg/day) has been identified that corrects Zn deficiency and can be safely used in a clinical trial; and (c) these findings support the rationale for screening older NH residents for Zn deficiency as part of a broader nutritional assessment. Although the current study was not powered to detect the secondary clinical outcomes, exploratory analyses revealed a statistically significant pattern of reduced self-reported cough, fever, and chills among Zn -supplemented participants across multiple biweekly timepoints. As these symptom data were self-reported and not clinician-confirmed, these findings should be interpreted with caution as preliminary evidence of a potential respiratory benefit rather than a confirmed clinical outcome. Nonetheless, these data provide a strong rationale for evaluating clinically confirmed respiratory outcomes in a larger, adequately powered trial.

This study has some limitations that should be considered when interpreting these findings. First, recruitment was conducted within a single geographic region (Boston area), which may limit generalizability to NH populations in other regions or healthcare systems. However, NH populations across the United States share broadly similar characteristics in terms of age, comorbidity burden, nutritional risk, and care structure (36). Further, our prior studies (11, 13), conducted over a 10-year period across multiple NHs in the Boston area, consistently report a similar prevalence of Zn deficiency, supporting the broader relevance of these findings. Notably, the racial composition of the present cohort differed from that of our earlier work; participants were predominantly Black (83–89% across study arms, Table 1), whereas prior cohorts from our group were predominantly Caucasian [94% in Meydani et al. (11); and ~71–76% in Prasad et al. (12); and 86–87% in Barnett et al. (13)]. This demographic shift extends the population in which Zn deficiency has been characterized by our group and supports the generalizability of these findings to a more racially diverse NH population. Second, the respiratory symptom data including cough and fever were self-reported by participants at biweekly intervals and were not objectively verified through clinical examination or diagnostic testing, introducing the possibility of recall or reporting bias; however, the use of frequent, standardized assessments over a 12-month period may help mitigate this concern and capture temporal trends in symptom reporting. Finally, this study relied on serum Zn concentration as the sole biomarker of Zn status. While serum Zn is endorsed by IZiNCG as the best available biomarker for assessing Zn deficiency risk and is the only Zn biomarker with widely accepted clinical cutoff values, it has recognized limitations as an individual-level marker, including reduced sensitivity to marginal Zn insufficiency. Future trials in this population should incorporate food frequency questionnaires to estimate the dietary Zn-phytate ratio, which may help clarify potential dietary contributors to Zn deficiency (37). Although phytate intake could influence Zn bioavailability, we do not consider it a likely explanation for the deficiency observed here given typical dietary patterns in NH settings.

## 5 Conclusion

This randomized, double-blind, placebo-controlled pilot trial demonstrated that 60 mg/day elemental Zn for 8 months achieved serum Zn adequacy (≥ 70 μg/dL) in 100% of Zn-deficient NH residents, with no adverse effects on copper status or other clinical chemistry parameters. Participants receiving the 30 mg/day dose did not achieve Zn adequacy in all participants over 12 months, supporting 60 mg/day as the optimal dose for a larger trial aimed at determining the clinical benefits of maintaining adequate Zn nutriture. Exploratory analyses also indicated a statistically significant reduction in self-reported respiratory symptoms, including cough, fever, and chills, among Zn -supplemented participants; as these symptom data were self-reported and not clinician-confirmed, this finding should be interpreted as preliminary rather than a confirmed clinical outcome. Taken together, with prior observations linking adequate Zn status to lower PNA incidence, these findings support conducting a fully powered randomized trial to evaluate the impact of Zn supplementation on PNA prevention in this population.

## Data Availability

The raw data supporting the conclusions of this article will be made available by the authors, without undue reservation.
