# Supplementary material for: Correcting zinc deficiency in nursing home residents: a randomized trial comparing 30 and 60 mg/day elemental zinc supplementation
Source: Front Nutr. 2026 Sep 30;13:1935770. doi: 10.3389/fnut.2026.1935770 (PMC13661384; doi:10.3389/fnut.2026.1935770)
Supplement: Supplementary file 1 [file Image_1.PDF]

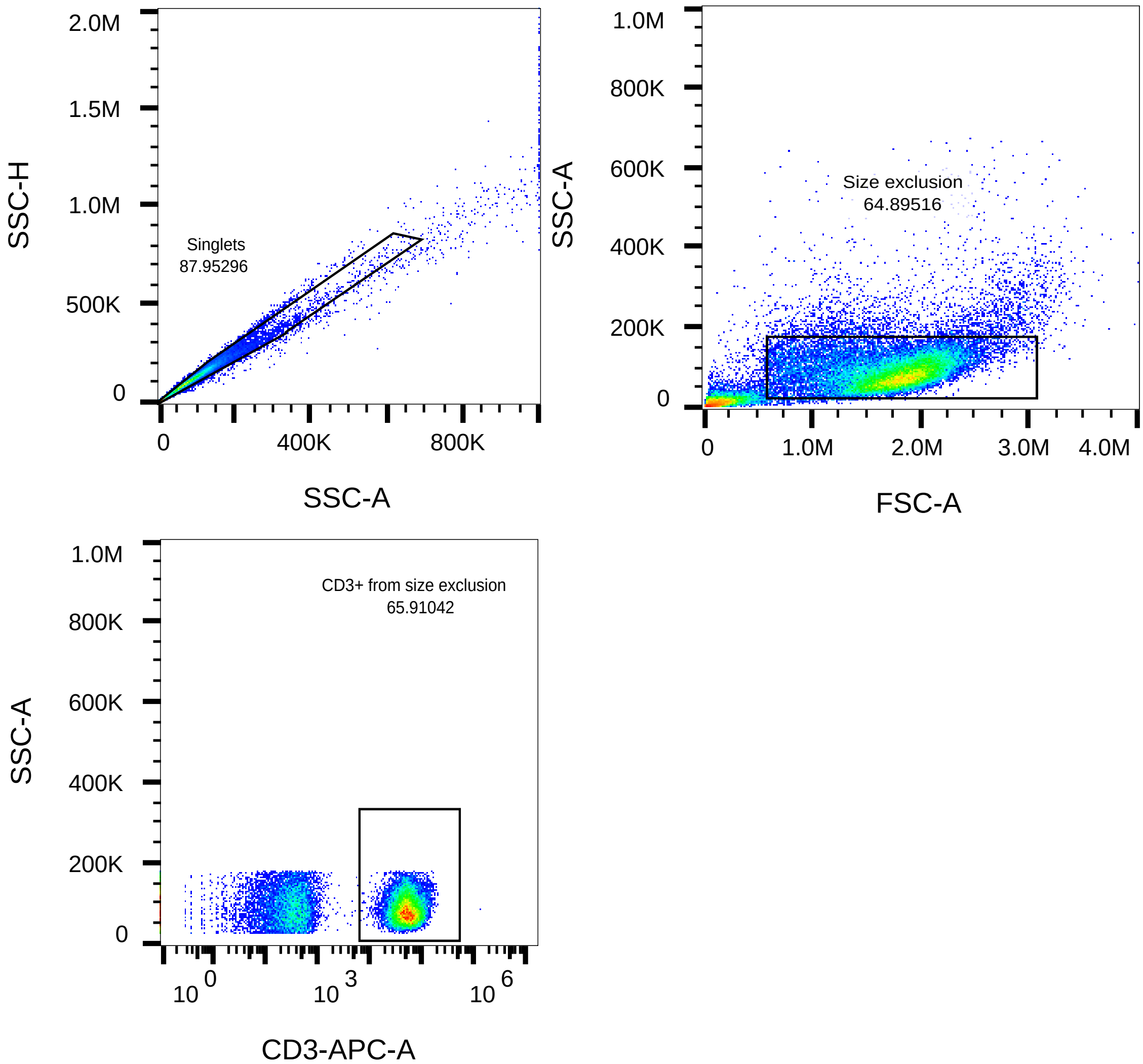

SSC-H
2.0M
1.5M
1.0M
500K
0
Singlets
87.95296
0
400K
800K
SSC-A
SSC-A
1.0M
800K
600K
400K
200K
0
Size exclusion
64.89516
0
1.0M
2.0M
3.0M
4.0M
FSC-A
SSC-A
1.0M
800K
600K
400K
200K
0
CD3+ from size exclusion
65.91042
10 0
10 3
10 6
CD3-APC-A
