# Supplementary material for: Correcting zinc deficiency in nursing home residents: a randomized trial comparing 30 and 60 mg/day elemental zinc supplementation
Source: Front Nutr. 2026 Sep 30;13:1935770. doi: 10.3389/fnut.2026.1935770 (PMC13661384; doi:10.3389/fnut.2026.1935770)
Supplement: Supplementary file 2 [file Image_2.PDF]

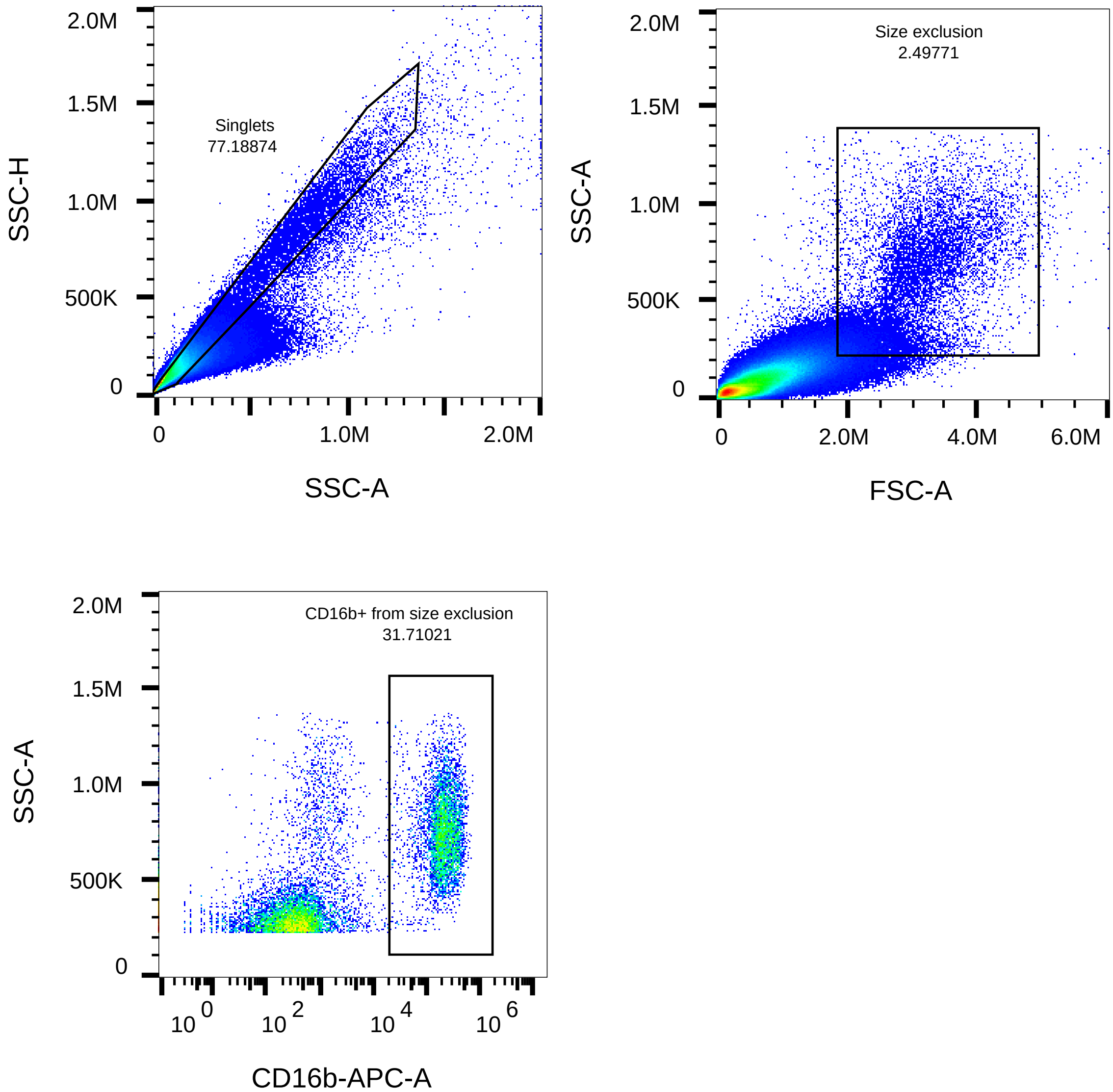

Singlets
77.18874
SSC-H
SSC-A
2.0M
1.5M
1.0M
500K
0
1.0M
2.0M
Size exclusion
2.49771
FSC-A
4.0M
6.0M
CD16b+ from size exclusion
31.71021
CD16b-APC-A
10 0
10 2
10 4
10 6
