# Supplementary material for: Correcting zinc deficiency in nursing home residents: a randomized trial comparing 30 and 60 mg/day elemental zinc supplementation
Source: Front Nutr. 2026 Sep 30;13:1935770. doi: 10.3389/fnut.2026.1935770 (PMC13661384; doi:10.3389/fnut.2026.1935770)
Supplement: Supplementary file 3 [file Image_3.PDF]

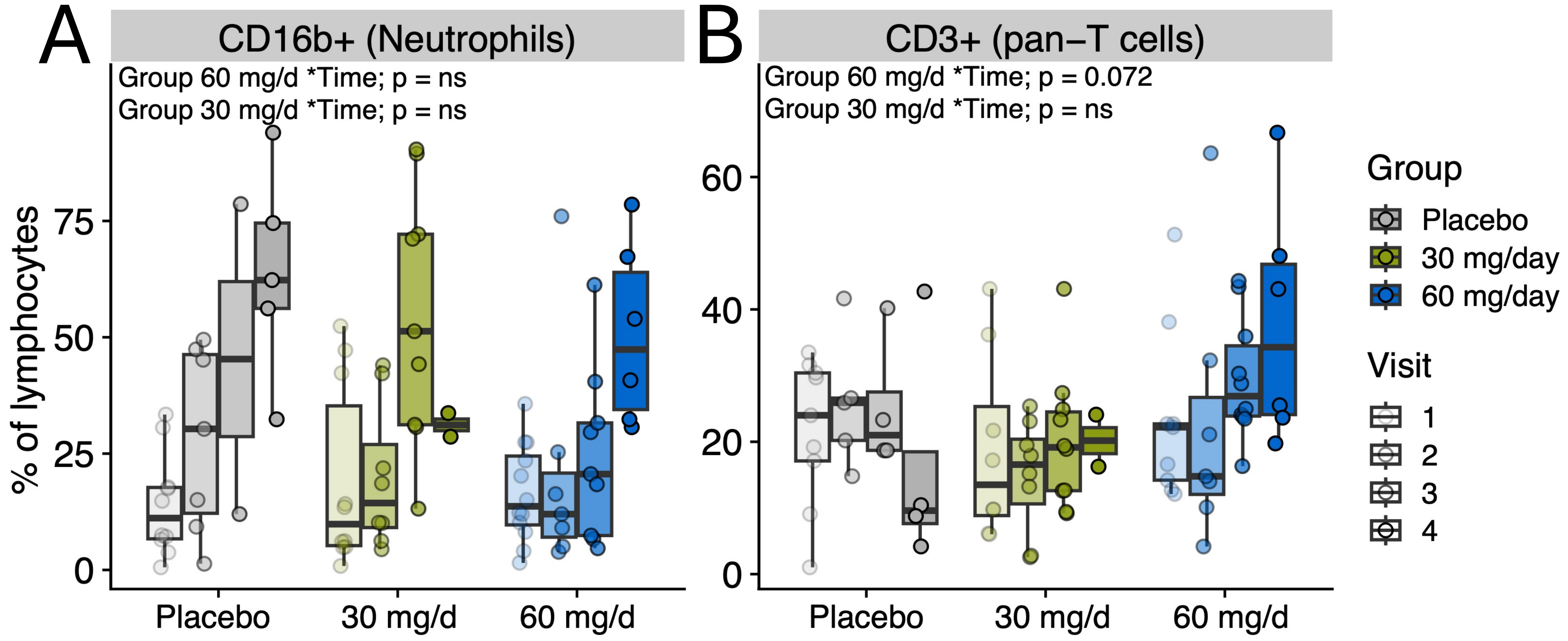

Supplementary Figure 3. Blood neutrophil and pan-T cell frequencies. **A)** There were no significant main effects of group or visit for T-cells. There was a trend ($p = 0.072$) toward a group-by-time interaction, driven by an increasing trajectory in the high-dose zinc group (60 mg/day) relative to placebo ($\beta = 5.57$, $p = 0.072$), suggesting a potential time-dependent effect of supplementation on T-cell frequency. The low-dose group did not differ from placebo in trajectory over time. **B)** Neutrophil frequencies did increase significantly during the study (Visit, $p < 0.05$), but there were no significant differences between groups. Linear mixed effects models were used to assess longitudinal changes in T-cell and Neutrophil frequencies across visits, accounting for repeated measures within participants. A p-value < 0.05 was considered significant, n = 2-12/group.
