# Supplementary material for: Correcting zinc deficiency in nursing home residents: a randomized trial comparing 30 and 60 mg/day elemental zinc supplementation
Source: Front Nutr. 2026 Sep 30;13:1935770. doi: 10.3389/fnut.2026.1935770 (PMC13661384; doi:10.3389/fnut.2026.1935770)
Supplement: Supplementary file 4 [file Table_1.DOCX]

**Supplementary Table 1: ½ RDA formulation**

| **Micronutrient** | **Chemical Form/Species** | **Amount per capsule** | **% DV** |
| --- | --- | --- | --- |
| Vitamin A | Vitamin A palmitate | 1333 IU | 44.4 |
| Vitamin C | Ascorbic acid (95%) | 40 mg | 44.4 |
| Vitamin D | Vitamin D (cholecalciferol) | 100 IU | 12.5 |
| Vitamin E | Vitamin E (Novatol HPMC) | 11.19 IU | 50.0 |
| Vitamin B1 | Thiamin mononitrate | 0.60 mg | 50.0 |
| Vitamin B2 | Riboflavin | 0.60 mg | 50.0 |
| Vitamin B3 | Niacinamide | 7.5 mg | 46.9 |
| Vitamin B6 | Pyridoxine HCl | 0.8 mg | 47.1 |
| Folate | Folic acid | 0.20 mg | 50.0 |
| Vitamin B12 | Vitamin B12 (1% on DCP) | 0.0012 mg | 50.0 |
| Copper | Copper gluconate | 0.8 mg | 88.9 |
| Iodine | Potassium iodide | 0.075 mg | 50.0 |
| Iron | Ferrous fumarate (95%) | 4 mg | 22.2 |
| Selenium | Selenomethionine (1%, Se 0.5% DCP) | 0.0275 mg | 50.0 |
| Zinc | Zinc gluconate | **Placebo: 5 mg, 30 mg-arm: 30 mg, 60 mg-arm: 60 mg** | 45.5,  272.7, 545.5 |

*%DV based on U.S. RDA (National Academy of Medicine DRIs), 2000-calorie reference diet.

*Note: All micronutrients besides zinc were provided at identical amounts and chemical form across all three study arms. Capsules were formulated to be visually indistinguishable across arms (identical size, appearance), with microcrystalline cellulose used as an inert filler to normalize total capsule weight across differing zinc doses.*

**Supplementary Table 2: Neutrophil-to-Lymphocyte Ratio (NLR) by Group and Visit**

| Group | Visit | n | Mean NLR (± SD) | Median NLR |
| --- | --- | --- | --- | --- |
| Placebo | Baseline | 35 | 2.19 (± 1.03) | 1.97 |
| Placebo | 4 months | 26 | 1.87 (± 0.72) | 1.74 |
| Placebo | 8 months | 26 | 1.80 (± 0.74) | 1.81 |
| Placebo | 12 months | 26 | 2.03 (± 1.19) | 1.73 |
| 30 mg/day | Baseline | 35 | 2.07 (± 0.92) | 1.85 |
| 30 mg/day | 4 months | 28 | 1.74 (± 0.62) | 1.75 |
| 30 mg/day | 8 months | 28 | 1.95 (± 1.18) | 1.83 |
| 30 mg/day | 12 months | 28 | 2.02 (± 1.13) | 1.76 |
| 60 mg/day | Baseline | 34 | 2.02 (± 0.98) | 1.80 |
| 60 mg/day | 4 months | 30 | 2.14 (± 0.90) | 2.02 |
| 60 mg/day | 8 months | 30 | 1.86 (± 1.27) | 1.62 |
| 60 mg/day | 12 months | 29 | 1.41 (± 0.58) | 1.42 |

NLR = neutrophil-to-lymphocyte ratio, calculated as absolute neutrophil count / absolute lymphocyte count. Mixed-effects linear regression (Time × Group, with participant as a random effect) revealed no significant main effect of time (p = 0.38) or Time×Group interaction for the 30 mg/day (p = 0.55) or 60 mg/day (p = 0.22) arms relative to placebo.
